# The Essential Role of Peptidylarginine Deiminases 2 for Cytokines Secretion, Apoptosis, and Cell Adhesion in Macrophage

**DOI:** 10.3390/ijms21165720

**Published:** 2020-08-10

**Authors:** Hui-Chun Yu, Chien-Hsueh Tung, Kuang-Yung Huang, Hsien-Bin Huang, Ming-Chi Lu

**Affiliations:** 1 Division of Allergy, Immunology and Rheumatology, Dalin Tzu Chi Hospital, Buddhist Tzu Chi Medical Foundation, Dalin, Chiayi 62130, Taiwan; junvsusagi@gmail.com (H.-C.Y.); dr5188@yahoo.com.tw (C.-H.T.); hky0919@yahoo.com.tw (K.-Y.H.); 2 School of Medicine, Tzu Chi University, Hualien City 97071, Taiwan; 3 Department of Life Science and Institute of Molecular Biology, National Chung Cheng University, Minxiong, Chiayi 62130, Taiwan; biohbh@ccu.edu.tw

**Keywords:** citrullination, macrophages, PADI2, inflammatory cytokines, adhesion, apoptosis

## Abstract

Objective: The study aims to investigate the functional roles of peptidylarginine deiminase 2 (PADI2) in macrophages. Methods: The clustered regularly interspaced short palindromic repeats (CRISPR)–CRISPR-associated protein-9 nuclease (Cas9) system was used to knockout PADI2 in U937 cells. U937 cells were introduced to differentiate macrophages and were stimulated with lipopolysaccharides (LPS). The protein expression of PADI2, PADI4, and citrullinated proteins were analyzed by Western blotting. The mRNA and protein levels of interleukin 1 beta (*IL-1β*), *IL-6*, and tumor necrosis factor-alpha (*TNF-α*) were analyzed using RT-PCR and ELISA, respectively. Cell apoptosis was analyzed using flow cytometry. Cell adhesion assay was performed using a commercially available fibrinogen-coated plate. Results: PADI2 knockout could markedly suppress the PADI2 protein expression, but not the PADI4 protein expression. PADI2 knockout decreased the protein levels of citrullinated nuclear factor κB (NF-κB) p65, but not those of citrullinated histone 3, resulting in the decreased mRNA expression levels of *IL-1β* and *TNF-α* in the U937 cells and *IL-1β* and *IL-6* in the differentiated macrophages and the macrophages stimulated with LPS. The cytokines levels of IL-1β, IL-6, and TNF-α were all dramatically decreased in the PADI2 knockout group compared with in the controls. PADI2 knockout prevented macrophages apoptosis via the decreased caspase-3, caspase-2, and caspase-9 activation. PADI2 knockout also impaired macrophages adhesion capacity through the decreased protein levels of focal adhesion kinase (FAK), phospho-FAK, paxillin, phospho-paxillin, and p21-activated kinase 1. Conclusion: This study showed that PADI2 could promote IL-1β, IL-6, and TNF-α production in macrophages, promote macrophage apoptosis through caspase-3, caspase-2, and caspase-9 activation and enhance cell adhesion via FAK, paxillin, and PAK1. Therefore, targeting PADI2 could be used as a novel strategy for controlling inflammation caused by macrophages.

## 1. Introduction

Peptidylarginine deiminases (PADIs) are a group of enzyme that converts peptidyl-arginine to peptidyl-citrulline, also called protein citrullination, in the presence of Ca^2+^ [1]. There are five members in the human PADI family, and each member has its own tissue distribution and substrate specificity [2]. PADIs and protein citrullination is known not only to contribute to the pathogenesis of several autoimmune diseases, such as rheumatoid arthritis (RA) and multiple sclerosis [1,3,4], but they were also recently found to facilitate cancer invasion and metastasis [5,6]. In human leukocytes, PADI2 and PADI4 are highly expressed [1]. Our previous study showed that the expression of PADI2 and PADI4 was remarkably increased during macrophage differentiation whereas the addition of lipopolysaccharides (LPS) increased the levels of citrullinated proteins. We further provided the evidence that PADI2 might play a critical role in the inflammatory response using plasmid-encoding short hairpin RNA-targeting PADI2 [7]. This result is consistent with that reported by Bawadekar et al., which demonstrated that PADI2-deficient mice showed a reduced joint inflammation in murine tumor necrosis factor-alpha (TNF-α)-induced arthritis [8].

Recently, clustered regularly interspaced short palindromic repeats (CRISPR) and the CRISPR-associated protein-9 nuclease (Cas9) system (CRISPR–Cas9 system), an effective way to edit genome [9], has become a powerful tool for investigating the biologic function of a specific gene [10]. We hypothesized that PADI2 is required for multiple domains of macrophage functions. Therefore, we used the CRISPR–Cas9 system to knockout PADI2 in macrophages and to evaluate the effects of PADI2 knockout on various functions of macrophages, including inflammation, cell survival, and adhesion capacity.

## 2. Results

### 2.1. Validation and Characterization of PADI2 Knockout U937 Cells

**Figure 1:**
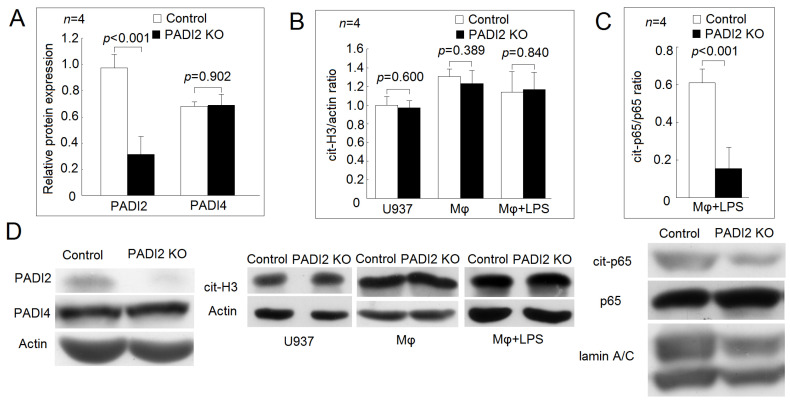
Validation of peptidylarginine deiminase 2 (PADI2) knockout U937 cells and their effects on histone H3 and nuclear factor kappa B (NF-κB) p65 citrullination. (**A**) Comparison of the relative protein expression levels of PADI2 and PADI4 with those of the control. The U937 cells were transfected with CRISPR–Cas9 plasmids containing gRNA that targets PADI2 or control CRISPR-Cas9 plasmids as the control group. The protein expression levels of PADI2 decreased dramatically, but those of PADI4 did not changed after PADI2 gene knockout (PADI2 knockout group). (**B**) Comparison of the citrullinated histone H3 (cit-H3)/actin ratios of the U937 cells, the differentiated macrophages, and the macrophages stimulated with 20 ng/mL lipopolysaccharides (LPS). The protein level of cit-H3 did not change in the U937 cells, the differentiated macrophages, and the macrophages stimulated with 20 ng/mL LPS for 24 h in the PADI2 knockout group. (**C**) Comparison of the citrullinated p65 subunit of NF-κB (cit-p65) ratios of the LPS-stimulated macrophages. The citrullinated protein was obtained from the nuclear extract of LPS-stimulated macrophages by immunoprecipitation using ACPAs-conjugated protein G Sepharose beads. The protein expression of the cit-p65 in the immunoprecipitates was then analyzed by Western blotting using anti-p65 antibodies as a probe. (**D**) Representative images showing the relative protein expression levels of PADI2 and PADI4; citrullinated histone H3 of the U937 cells, the differentiated macrophages, and the macrophages stimulated with LPS and cit-p65 in the immunoprecipitates of the LPS-stimulated macrophages in the PADI2 knockout group and the control group.

We confirmed the protein expression of PADI2 was dramatically decreased after gene knockout using the CRISPR–Cas system (Figure 1A). PADI2 and PADI4 are homologous in their structures and amino acid sequences in human [2]. We found that the protein expression of PADI4 did not change in the U937 cells after PADI2 knockout compared with those in the controls (Figure 1A). Next, we analyzed the protein levels for cit-H3. In the U397 cells, the differentiated macrophages, and the macrophages stimulated with LPS, the protein levels of cit-H3 were not different between the PADI2 knockout group and the controls (Figure 1B). Sun et al. reported that PADI4 could citrullinate nuclear factor κB (NF-κB) p65 and enhance its nuclear translocation and transcriptional activity [11]. We found that the protein levels of the citrullinated p65 were decreased in the nuclear extract of macrophages stimulated with LPS in the PADI2 knockout group compared with in the controls (Figure 1C,D).

### 2.2. Effects of PADI2 Knockout on Proinflammatory Cytokines Expression and Secretion

**Figure 2:**
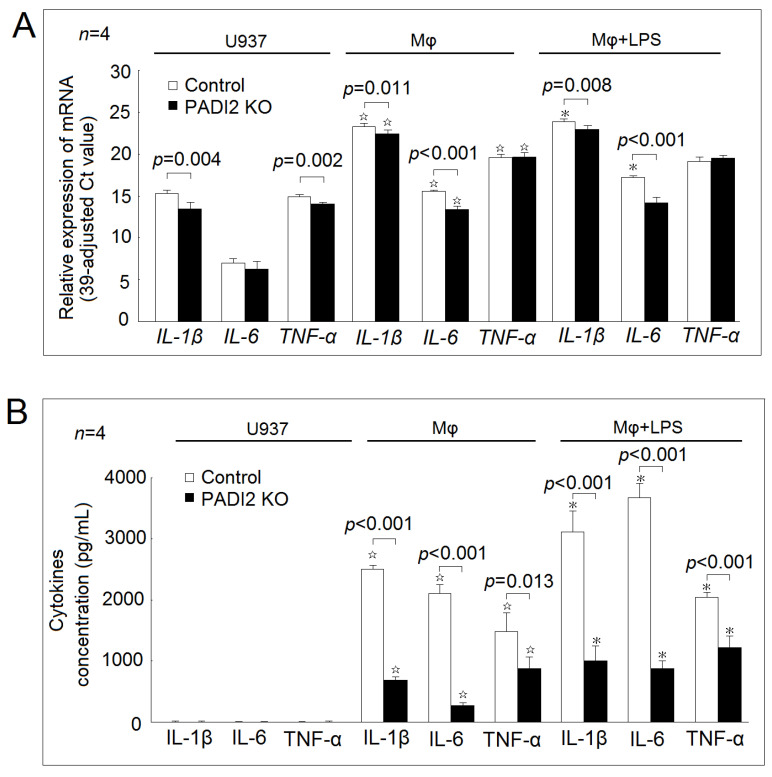
Effects of PADI2 knockout on inflammatory cytokines expression and secretion. (**A**) The mRNA expression levels of *IL-1β*, *IL-6*, and tumor necrosis factor-alpha (*TNF-α*) in the U937 cells, the differentiated macrophages, and the macrophages stimulated with LPS in the PADI2 knockout group compared with those in the controls. (**B**) The cytokines secretion levels of IL-1β, IL-6, and TNF-α in the U937 cells, the differentiated macrophages, and the macrophages stimulated with LPS transfected in the PADI2 knockout group compared with those in the control group. ^☆^ *p* < 0.05 compared with the U937 cells; * *p* < 0.05 compared with the differentiated macrophages.

In Figure 2A, we found that the gene expression levels of *IL-1β*, *IL-6*, and *TNF-α* were increased in the differentiated macrophages compared with in the U937 cells in both the PADI2 knockout group and the control group. The addition of LPS further increased the mRNA expression levels of *IL-1β* (23.9 ± 0.2 vs. 23.3 ± 0.3; *p* = 0.017) and *IL-6* (17.2 ± 0.2 vs. 15.5 ± 0.1; *p* < 0.001), but not those of *TNF-α* (19.1 ± 0.5 vs. 19.6 ± 0.3; *p* = 0.135) in the control group. The addition of LPS did not affected the mRNA expression levels of f *IL-1β*, *IL-6*, and *TNF-α* in the PADI2 knockout group.

In the U937 cells, the PADI2 knockout decreased the gene expression levels of *IL-1β* and *TNF-α* compared with those in the controls. In the differentiated macrophages and the macrophages stimulated with LPS, the PADI2 knockout decreased the gene expression levels of *IL-1β* and *IL-6* compared with those in the controls.

In the U937 cells, the secretion levels of IL-1β, IL-6, and TNF-α were very low in both the PADI2 knockout group and the control group (Figure 2B). In both groups, the differentiated macrophages secreted the increased levels of IL-1β, IL-6, and TNF-α compared with the U937 cells. The addition of LPS further increased the secretion levels of IL-1β, IL-6, and TNF-α in both groups.

In the differentiated macrophages and the macrophages stimulated with LPS, PADI2 knockout significantly decreased the cytokine secretion levels of IL-1β, IL-6, and TNF-α compared with in the controls.

### 2.3. Effects of PADI2 Knockout on Cell Apoptosis and Adhesion

**Figure 3:**
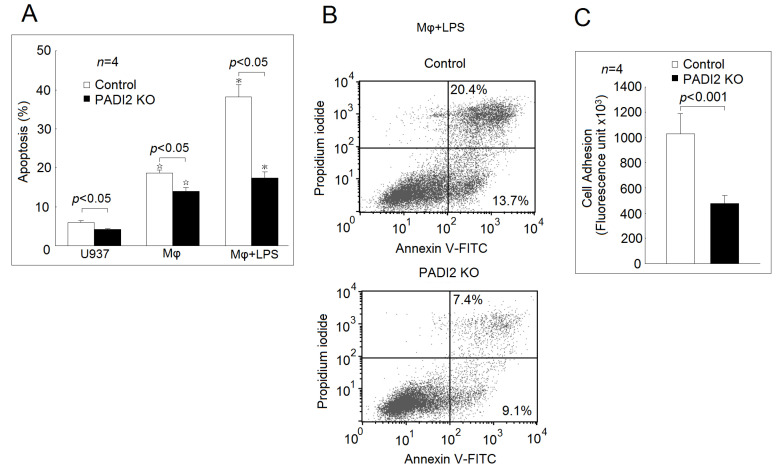
Effects of PADI2 knockout on macrophages apoptosis and adhesion. (**A**) The apoptosis rates of the U937 cells, the differentiated macrophages, and the macrophages stimulated with LPS for 24 h in the PADI2 knockout and control groups. The apoptotic rates of these cells were measured using flow cytometry analysis. The cells apoptosis was defined as % of annexin V staining. (**B**) Comparison of cell apoptosis in the macrophages stimulated with LPS in the PADI2 knockout group and the control group. It was shown than cell apoptosis in the macrophages stimulated with LPS in the PADI2 knockout group was decreased compared with in the control group. (**C**) The adhesion ability in the differentiated macrophages from the PADI2 knockout group and the control group using a fibrinogen-coated plate. ^☆^ *p* < 0.05 compared with the U937 cells; * *p* < 0.05 compared with the differentiated macrophages.

As expected, the apoptotic rate was significantly elevated in the differentiated macrophages compared with in the U937 cells. The addition of LPS further increased the apoptotic rate of the macrophages in both the PADI2 knockout group and the control group (Figure 3A,B). The PADI2 knockout group significantly decreased the apoptotic rates in the U937 cells, the differentiated macrophages, and the macrophages stimulated with LPS compared with the control group. We also noticed that the PADI2 knockout macrophages were more easily detached during trypsinization compared with the controls. Stefanelli et al. showed that protein citrullination could alter focal adhesion stability [12]. Therefore, we speculated that PADI2 knockout might also impair the macrophage adhesion. Using a commercially available fibrinogen-coated plate, we found that the PADI2 knockout group had impaired cell adhesion ability compared with the control group (Figure 3C).

### 2.4. Effecst of PADI2 Knockout on the Protein Expression of Caspases

**Figure 4:**
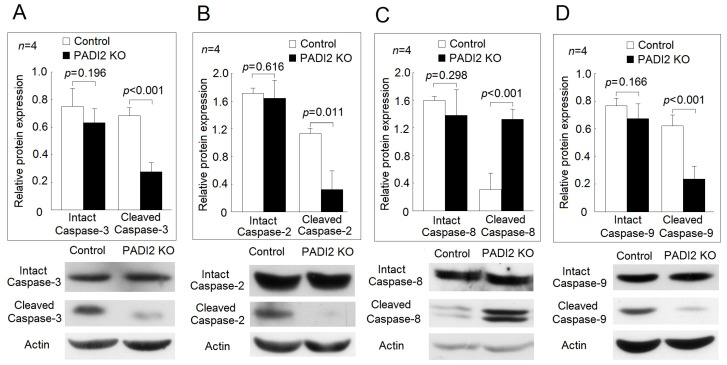
Effects of PADI2 knockout on the relative expression levels of the activated caspase-3, -2, -8, and -9. In the differentiated macrophages stimulated with LPS, the protein expression levels of the intact caspases and the cleaved caspases were performed by Western blotting in the PADI2 knockout group and the control group. The relative protein expression was defined as the ratio of intact caspases or cleaved caspases to the actin band intensity: (**A**) caspase-3; (**B**) caspase-2; (**C**) caspase-8; (**D**) caspase-9.

We further investigated the effects of PADI2 knockout on the activation of different caspases. In the macrophages stimulated with LPS, we found that the protein levels of the cleaved caspase-3, but not those of the intact caspase-3, were decreased in the PADI2 knockout group compared with in the controls. Among the initiator caspases [13], we found that the protein levels of the cleaved caspase-2 and the cleaved caspase-9, but not those of the intact caspase-2 and the intact caspase-9, were also significantly decreased in the PADI2 knockout group compared with in the controls (Figure 4). In contrast, the protein levels of the cleaved caspase-8, but not those of the intact caspase-8, were significantly elevated in the PADI2 knockout group compared with in the controls.

### 2.5. Effecst of PADI2 Knockout on the Protein and mRNA Expression of Adhesion-Related Genes

**Figure 5:**
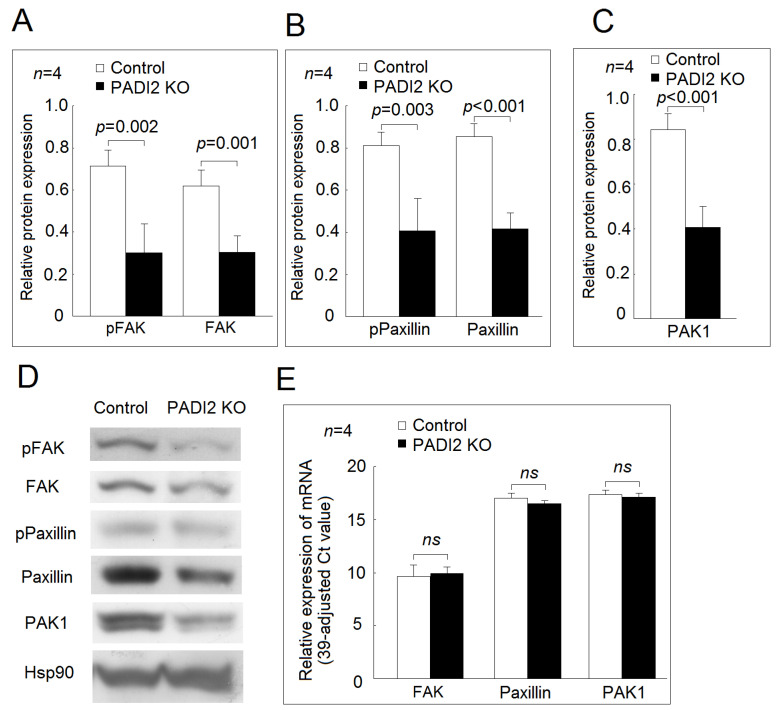
Effects of PADI2 knockout in the protein and mRNA expression of focal adhesion kinase (*FAK*), *paxillin*, and *PAK1* in the U937 cells: (**A**) comparison of the relative protein expression levels of FAK and phospho-FAK in the PADI2 knockout group and the control group; (**B**) comparison of the relative protein expression levels of paxillin and phospho-paxillin in the PADI2 knockout group and the control group; (**C**) comparison of the relative protein expression levels of PAK1 in the PADI2 knockout group and the control group; and (**D**) Representative images showing the relative protein expression levels of FAK, paxillin, and PAK1 in the PADI2 knockout group and the control group; (**E**) the relative mRNA expression levels of *FAK*, *paxillin*, and *PAK1* in the U937 cells. In the U937 cells, the protein expression levels of the cell adhesion-related proteins, including FAK, phospho-FAK, paxillin, phospho-paxillin, and PAK1, were performed by Western blotting in the PADI2 knockout group and the control group. The relative protein expression was defined as the ratio of specific protein/hsp90 band intensity.

**Figure 6:**
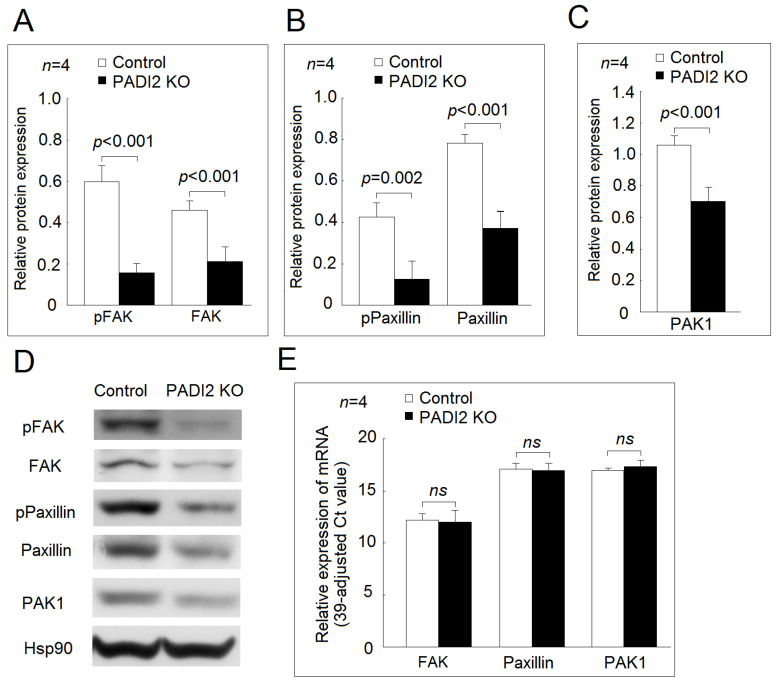
Effects of PADI2 knockout in the protein and mRNA expression of *FAK, paxillin*, and *PAK1* in the differentiated macrophages: (**A**) comparison of the relative protein expression levels of FAK and phospho-FAK in the PADI2 knockout group and the control group; (**B**) comparison of the relative protein expression levels of paxillin and phospho-paxillin in the PADI2 knockout group and the control group; (**C**) comparison of the relative protein expression levels of PAK1 in the PADI2 knockout group and the control group; and (**D**) Representative images showing the relative protein expression levels of FAK, paxillin, and PAK1 in the PADI2 knockout group and the control group; (**E**) the relative mRNA expression levels of *FAK*, *paxillin*, and *PAK1*. In differentiated macrophages, the protein expression levels of the cell adhesion-related proteins including FAK, phospho-FAK, paxillin, phospho-paxillin, and PAK1 were performed by Western blotting in the PADI2 knockout group and the control group. The relative protein expression was defined as the ratio of a specific protein/hsp90 band intensity.

In the U937 cells and the differentiated macrophages, we found that the protein expression levels of phospho-FAK, FAK, phospho-paxillin, paxillin, and PAK1 were all decreased in the PADI2 knockout group compared with in the control group (Figure 5 and Figure 6). For the gene expression of the adhesion-related proteins, we found that there were no statistically significant differences in the mRNA expression of *FAK*, *paxillin*, or *PAK1* between the PADI2 knockout group and the control group in the U937 cells and the differentiated macrophages.

## 3. Discussion

Vossenaar et al. [14], Hojo-Nakashima et al. [15], and our previous study [7] have shown that PADI2 protein levels increased during the macrophage differentiation, resulting in increasing protein citrullination. In the current study, we demonstrated that PADI2 is essential for macrophage proinflammatory cytokine secretion, cell adhesion, and apoptosis using CRISPR/Cas9-mediated knockout of PADI2. As for the target proteins of PADI2, we found the histone H3 citrullination did not change in the U937 cells, the differentiated macrophages, or the macrophages stimulated with LPS in the PADI2 knockout group compared with in the control group. Darrah et al. reported that histone H3 was prone to be citrullinated by PADI4, which might explain our finding [16]. Different antibodies used in diffident studies might detect different epitopes, which could affect the results of histone H3 citrullination. However, we demonstrated that the protein level of citrullinated NF-κB p65 was decreased in the PADI2 knockout group compared with in the controls. Sun et al. showed that the citrullination of NF-κB p65 could enhance *IL-1β* and *TNF-α* expression [11]. Our result also showed that the expression levels of *IL-1β* and *TNF-α* were indeed decreased in the U937 cells after PADI2 knockout. The differentiation of macrophage and further stimulation with LPS could decrease the *IL-1β* and *IL-6* expression in the PADI2 knockout group compared with in the controls. Most importantly, we found that the IL-1β, IL-6, and TNF-α concentrations in the culture soup were dramatically decreased in the PADI2 knockout group compared with in the controls. In addition, Sun et al. showed citrullinated NF-κB p65 was mediated by PADI4 using HeLa cells and neutrophils, which expressed high levels of PADI4 compared with those of monocytes and lymphocytes [6]. In the current study, we used U937 cells, a representative cell line for human monocytes. We demonstrated that PADI2 was also required to citrullinated NF-κB p65 in macrophages upon LPS stimulation. Lee et al. showed that PADI2 could interact with an inhibitor of nuclear factor kappa-B kinase subunit gamma (IKKγ) to suppress NF-κB activity using a murine cell line [17]. However, our results suggested that NF-κB activity decreased in PADI2 knockout U937 cells from a decreased expression of proinflammatory cytokines. Different cell lines used in different studies might explain these variations. Mishra et al. demonstrated that PADI2 and PADI4 activity in macrophages were required for inflammasome assembly and IL-1β release in a murine model [18], which is consistent with our findings. Since proinflammatory cytokines including IL-1β, IL-6, and TNF-α play a critical role in the immunopathogenesis of RA [19,20] and increased PADIs activities have been documented in patients with RA [21], targeting PADIs would be a novel strategy for RA treatment.

We found that PADI2 knockout significantly decreased the apoptotic rates in the U937 cells, the differentiated macrophages, and the macrophages stimulated with LPS. Liu et al. demonstrated the overexpression of PADI4-induced cell apoptosis in human leukemia (HL)-60 cells and human acute T leukemia Jurkat cells [22]. These findings suggested that both PADI2 and PADI4 could promote apoptosis. For the detail mechanism of cell apoptosis, Liu et al. found that PADI4 induced apoptosis mainly through cell cycle arrest and a mitochondria-mediated pathway [22]. Our study showed that PADI2 could activate caspase-2 and -9, leading to the activation of capases-3. Further studies are needed to clarify their molecular mechanisms.

We noted that the inhibition of PADI2 impaired the protein expression of phospho-FAK, FAK, phospho-paxillin, paxillin, and PAK1, resulting in impaired cell adhesion ability in macrophages. We could not detect the phosphorylation of PAK1, which was also not detected in macrophages in a previous study [23]. PAK1 belongs to one of the members of the PAKs family, which plays an important role in cell motility [24], and the phosphorylation of PAK1 is critical for cell migration instead of adhesion [25]. We noted that the protein levels, but not mRNA expression levels of FAK, paxillin, and PAK1, were decreased in the PADI2 knockout group compared with in the controls. We speculated that the citrullination of proteins might accelerate their degradation by changing their binding affinity to proteasome [7,26] or altered protein structure [27], and further studies are needed.

In conclusion, our study showed that PADI2 is essential for the multiple functions of macrophages in enhancing inflammatory cytokines production through the citrullination of NF-κB p65, promoting cell apoptosis with capase-2, -3, and -9 activation, and facilitating cell adhesion via FAK, paxillin, and PAK1.Targeting PADI2 could be a novel strategy for controlling inflammation triggers by macrophages.

## 4. Material and Methods

### 4.1. Purification of Anticitrullinated Protein Antibodies (ACPA) from Pooled ACPA(+) Sera in Patients with RA

The study protocol was approved by the institutional review board of Dalin Tzu Chi Hospital, Buddhist Tzu Chi Medical Foundation (No. B10902001, 1 April 2020). The study was performed in accordance with the Declaration of Helsinki. Serum samples from ACPA-positive RA patients with high concentration of ACPAs (>340 IU/mL) and aged 20 years and above, which fulfilled the 2010 American College of Rheumatology (ACR)/European League Against Rheumatism (EULAR) criteria [28], were detected using an ELISA kit (Pharmacia Diagnostics AB, Uppsala, Sweden), collected and pooled. ACPAs were purified according to the method described previously [29]. In brief, the pooled sera containing high concentration of ACPAs from patients with RA were purified by affinity chromatography using an ÄKTA purifier 10 (GE Healthcare, Little Chalfont, UK) with UV detection at 280 nm for the collection of the desired fractions.

### 4.2. Preparation of PADI2 Knockout U937 Cells

U937 cells were purchased from the European Collection of Cell Cultures (Salisbury, UK) and electroporated with control CRISPR–Cas9 plasmids or CRISPR–Cas9 plasmids containing gRNA that targeted PADI2 (Santa Cruz biotechnology, Dallas, TX, USA) using the Gene Pulser MXcell electroporation system (Bio-Rad Laboratories, Hercules, CA, USA) under the condition of voltage 210 V and capacitance 960 μF. The cells were then cultured in Iscove’s Modified Dulbecco’s medium (IMDM) (Invitrogen, Carlsbad, CA, USA) with 10% fetal bovine serum (Invitrogen, Carlsbad, CA, USA) with 0.3 μg/mL puromycin (Sigma-Aldrich, St. Louis, MO, USA). After drug selection, the surviving cells were isolated and validated by Western blotting. The U937 cells was then introduced to differentiate with “differentiated macrophages” by coculturation with 500 ng/mL phorbol 12-myristate 13-acetate (PMA, Sigma-Aldrich, St. Louis, MO, USA) at 37 °C in a humidified atmosphere containing 5% CO_2_ for 48 h. The differentiated macrophages were cocultured with LPS (20 ng/mL, Sigma-Aldrich, St. Louis, MO, USA) for 24 h at 37 °C in a humidified atmosphere containing 5% CO_2_. The culture supernatants were then collected and stored at −80 °C for ELISA.

### 4.3. ELISA

The concentrations of cytokines in the culture supernatants were determined using the respective ELISA kits (BD Biosciences, Franklin Lakes, NJ, USA) according to the manufacturer’s specification.

### 4.4. Flow Cytometry Analysis

Cell apoptosis was determined by double staining with the FITC-annexin V and propidium iodide (PI) kit (BD Biosciences, Franklin Lakes, NJ, USA) in cells analyzed by flow cytometry (FACScan, Becton Dickinson, Franklin Lakes, NJ, USA) using Lysis II software.

### 4.5. Cell Adhesion Assay

Cell adhesion assay was performed using a CytoSelect™ Cell Adhesion Assay Kit (Cell Biolabs, San Diego, CA, USA), which used a fibrinogen-coated plate, according to the manufacturer’s protocol with modifications. U937 cells cocultured with 500 ng/mL PMA for 24 h (7.5 × 10^4^ per well) were added and incubated with 500 ng/mL PMA at 37 °C for 24 h in a humidified atmosphere containing 5% CO_2_. Lysis buffer/CyQuant^®^ GR dyes (200 μL) were added and incubated for 20 min at room temperature with shaking. Finally, the mixture was transferred to a 96-well plate and analyzed using an ELISA microplate reader (Anthos Zenyth 3100, Cambridge, UK) with two separate readings (480 and 520 nm).

### 4.6. Preparation of the Cell Nuclear Protein Extract

Cells were lysed with 1% NP-40 (Sigma-Aldrich, St. Louis, MO, USA) in the presence of a proteinase inhibitor cocktail (Sigma-Aldrich, St. Louis, MO, USA) and a phosphatase inhibitor cocktail (Thermo Fisher Scientific, Waltham, MA, USA). A nuclear extract was prepared using a Nuclear Extract Kit (Active Motif, Carlsbad, CA, USA) according to the manufacturer’s protocol. The protein concentrations of these samples were measured using the Bradford method. The culture supernatants were collected and stored at −80 °C for further analysis.

### 4.7. Immunoprecipitation of Citrullinated Protein

The nuclear extract from macrophages stimulated by LPS was immunoprecipited by ACPAs-conjugated protein G Sepharose beads (Protein G Immunoprecipitaton kits, Sigma-Aldrich, St. Louis, MO, USA) according to the manufacturer’s instruction. Then, protein molecules in the supernatant (immunoprecipitates) were then analyzed by Western blotting using anti-p65 antibodies as a probe.

### 4.8. Western Blotting Analysis

Cell lysate was electrophoresed and transferred to a polyvinyllidene difluoride (PVDF) sheet (Sigma-Aldrich, St. Louis, MO, USA). The membranes were nonspecifically blocked in a 1% skim milk solution and incubated with primary antibodies for PADI2 (ab56928), PADI4 (ab128086), and citrullinated histone 3 (cit-H3; ab5103), caspase-2 (ab179520) (Abcam, Cambridge, UK), caspase-3 (9662), cleaved caspase-3 (9661), caspase-8 (9746), cleaved caspase-8 (9496), caspase-9 (9502), cleaved caspase-9 (7237), p21-activated kinase 1 (PAK1) (2602), focal adhesion kinase (FAK) (3285), phospho-FAK(8556), paxillin (12065), phospho-paxillin (2541), p65 (8242), and heat shock protein 90 (hsp90) (4874) (Cell Signaling Technology, Danvers, MA, USA) followed by the respective HRP-conjugated secondary antibodies (Jackson ImmunoResearch Laboratories, West Grove, PA, USA). Blotting was visualized by chemiluminescence reaction (ECL; GE Healthcare, Little Chalfont, UK). The respective band intensities were measured using ImageJ (version 1.42; http://rsb.info.nih.gov/ij).

### 4.9. Measurement of Cytokine Expression Levels by RT-PCR

Total RNA was extracted using a Quick-RNA MiniPrep kit (Zymo Research, Irvine, CA, USA) according to the manufacturer’s protocol. RNA concentrations were quantified using a spectrophotometer (NanoDrop 1000, Thermo Fisher Scientific, Waltham, MA, USA). The mRNA expression levels of interleukin 1 beta (*IL-1β*), *TNF-α*, *IL-6*, *FAK*, *paxillin*, and *PAK1* were quantified by RT-PCR by a one-step RT-PCR kit (TaKaRa, Shiga, Japan) with an ABI Prism 7500 Fast Real-Time PCR system (Applied Biosystems, Waltham, MA, USA) as previously described [30]. The relative expression levels of mRNA were defined by the following equation: (39—threshold cycle (Ct) after being adjusted by the expression of 18S ribosomal RNA).

### 4.10. Statistical Analysis

Data were expressed as mean ± standard deviation. Statistical significance was assessed by the Mann-Whitney U test. All statistical analyses were performed using Stata/SE version 8.0 for Windows (StataCorp, College Station, TX, USA). Two-tailed *P* values less than 0.05 were considered significant. The datasets analyzed during the current study are available from the corresponding author on reasonable request.

## Abbreviations

PADI	peptidylarginine deiminases
IL	interleukin
TNF-α	tumor necrosis factor-alpha
ACPAs	anticitrullinated protein antibodies
PMA	phorbol 12-myristate 13-acetate
LPS	lipopolysaccharides
CRISPR	clustered regularly interspaced short palindromic repeats
Cas9	CRISPR-associated protein-9 nuclease
RT-PCR	real-time reverse transcription-polymerase chain reaction
ELISA	enzyme-linked immunosorbent assay
NF-κB	nuclear factor kappa-light-chain-enhancer of activated B cells
PAK1	p21-activated kinase 1
FAK	focal adhesion kinase
SDS-PAGE	sodium dodecyl sulfate polyacrylamide gel electrophoresis
HRP	horseradish peroxidase
